# Mapping reviews, scoping reviews, and evidence and gap maps (EGMs): the same but different— the “Big Picture” review family

**DOI:** 10.1186/s13643-023-02178-5

**Published:** 2023-03-15

**Authors:** Fiona Campbell, Andrea C. Tricco, Zachary Munn, Danielle Pollock, Ashrita Saran, Anthea Sutton, Howard White, Hanan Khalil

**Affiliations:** 1 Population Health Sciences Institute, Newcastle University, Newcastle, UK; 2 Knowledge Translation Program of the Li Ka Shing Knowledge Institute, St. Michael’s Hospital, University of Toronto in the Dalla Lana School of Public Health & Institute of Health Policy, Management, and Evaluation, Toronto, Canada; 3 JBI, Faculty of Health and Medical Sciences, University of Adelaide, Adelaide, Australia; 4 International Development Coordinating Group, Campbell Collaboration, Oslo, Norway; 5 ScHARR, University of Sheffield, Sheffield, United Kingdom; 6 Evaluation and Evidence Synthesis, Global Development Network, New Delhi, India; 7 School of Psychology and Public Health, Department of Public Health, La Trobe University, Melbourne, Australia

## Abstract

Scoping reviews, mapping reviews, and evidence and gap maps are evidence synthesis methodologies that address broad research questions, aiming to describe a bigger picture rather than address a specific question about intervention effectiveness. They are being increasingly used to support a range of purposes including guiding research priorities and decision making. There is however a confusing array of terminology used to describe these different approaches. In this commentary, we aim to describe where there are differences in terminology and where this equates to differences in meaning. We demonstrate the different theoretical routes that underpin these differences. We suggest ways in which the approaches of scoping and mapping reviews may differ in order to guide consistency in reporting and method. We propose that mapping and scoping reviews and evidence and gap maps have similarities that unite them as a group but also have unique differences. Understanding these similarities and differences is important for informing the development of methods used to undertake and report these types of evidence synthesis.

## Introduction

Evidence synthesis(defined broadly as the rigorous collation, evaluation and analysis of literature, studies, and reports) is increasingly viewed as critical to inform decision making in policy and practice. Over the past three decades, as various methods of evidence synthesis have emerged and evolved, the systems and labels used to categorize different review types have proliferated. A recent catalog of evidence synthesis approaches and terms identified 48 distinct review types [1]. Moher et al. (2015) [2], describes them as a “family” of evidence synthesis products that have arisen in response to policymakers and other stakeholders needs for diverse forms of information. This growth reflects the increased value placed on evidence synthesis to inform decision making, and we now see evidence synthesis used to address a broader range of research questions beyond effectiveness, along with tailored approaches (in terms of methods and products) to evidence synthesis as appropriate for different research needs, purposes, situations, and audiences [3].

Examples of approaches that are increasingly seen in the published literature are scoping reviews, mapping reviews, and evidence and gap maps (EGMs). Scoping reviews, mapping reviews, and EGMs are relatively new approaches that rarely appeared before 2009 [4, 5]. Scoping reviews, evidence maps, and evidence and gap maps have been grouped together as “Big Picture” approaches due to their shared purpose and approaches. These Big Picture reviews can be contrasted with systematic reviews (addressing interventions, diagnostic test accuracy, prognosis, etc.) as they have a broader scope as compared to the (normally) narrower scope of classic systematic reviews. There have been consistent yearly increases in the publication of scoping, mapping, and evidence and gap maps [6]. Despite this, there remains confusion as to their application, meaning, and whether differences exist between them. This commentary aims to clarify these approaches, identify any differences between them, and provide recommendations for reviewers.

### Terminology matters

This growing and evolving family of evidence synthesis types presents some challenges [7].

Firstly, there is the challenge of choosing the correct approach, particularly when terms are used inconsistently in the literature. The selection of an appropriate review approach will ensure the correct methods are employed using the appropriate standards for both its conduct and reporting. Indexing and wider dissemination can be challenging for researchers when there is ambiguity in terms [8, 9].

### Scoping reviews and mapping reviews—how are they used in the literature

Scoping reviews, mapping reviews, and evidence maps are terms that are not used consistently in the literature, with different terms used to describe similar approaches and review objectives. The same term is also used to describe different approaches and review objectives. Within the published literature, the terms scoping reviews and mapping reviews appear to be used in three different ways. Firstly, the terms “mapping” and “scoping” reviews are used interchangeably, referring to the same type of review methodology [5, 6, 10]. This approach is also one that is used in the PRISMA Extension for Scoping Reviews (PRISMA-ScR) [11], providing guidance to inform reporting standards [12]. This may therefore have been influential in increasing the use of the term scoping review over the use of the term mapping review. Examination of published reviews does not reveal differences in method between these approaches (Campbell et al., 2022 publication in press).

Secondly, we see the terms used as complementary to the other. Some definitions tend to use the terms in a way which suggest that mapping is a specific approach to scoping—or vice versa. For example, “scoping reviews can usefully map the evidence in a number of ways” [13] and “scoping reviews are a way of mapping the key concepts” [14]. Lukersmith et al. (2016) [15] and Fernadez-Sotos et al. (2019) [16] suggest that the term map is a descriptive term used to describe one of the purposes of the scoping review. A mapping review may also scope the literature. It has also been suggested that when the term mapping is included in the description of the method that the review will incorporate a geographical mapping exercise or charting of the data in a tabular or any other visual format that can plot or portray the data.

Finally, we see scoping and mapping used to describe different types of evidence synthesis, and a distinction is made between mapping and scoping reviews [1, 17]. These authors suggest that scoping reviews are “preliminary assessment of potential size and scope of available research literature which aims to identify nature and extent of research evidence (usually including ongoing research)”. It also is a term that has emerged within the systematic review field to describe the preliminary work undertaken with information specialists in planning the review, by getting a sense of the size of the literature, to identify key terms and theories and potentially clinical experts [18]. Within these definitions, mapping reviews are distinguished from a scoping review because the subsequent outcome may involve either further review work or primary research and this outcome is not known beforehand. For the purpose of this paper, we will refer to these as a scoping exercise instead of a formal scoping review methodology. Scoping exercises within this definition would not usually be regarded as a final output in their own right, primarily because of limitations in their rigor mean that they hold the potential for bias.

Gough et al. (2012) [19] suggest that the term scoping review often describes a more rapid, and so usually non-systematic, approach to describing the nature of the literature on a topic area, sometimes as part of planning for a systematic review compared with a standard systematic review. It is also important to note that there are published rapid scoping reviews where streamlined methods are used, but transparency and rigor are maintained to produce quicker results for decision-making purposes. Examples of these types of rapid scoping reviews include rapid responses to policy questions during the COVID-19 pandemic [20, 21].

An alternative view of the difference comes from Bragge et al. (2011) [22] who suggests that a scoping review is distinguished from mapping by the inclusion of research results in the description of relevant evidence, whereas maps simply describe what is there without collating and summarizing the results of the studies.

So, even where the types of products are seen as different, there is not a consistent approach in this difference. Nevertheless, understanding why they are considered different is important in considering what is lost, in terms of an apt descriptor, if the terms are amalgamated and used interchangeably.

### Historical origins

One reason that the terms scoping and mapping have emerged to describe two similar methodological approaches addressing broad types of research questions lies in the academic traditions from which they derive and the epistemological foundations upon which these are built. Scoping reviews and scoping review methodological guidance [12] tends to cite the framework defined by Arksey and O’Malley (2005) [23] and later enhancements by Levac et al. (2010) [24]. These approaches have their roots in sociological sciences. In contrast, the term evidence mapping was first used by Katz et al. (2003) [25] and has roots in the natural sciences. This was the term adopted by the EPPI Center in an early publication of a mapping review and is the term used by the Center for Environmental Evidence for the environmental sciences. The approach to evidence mapping accompanied by a visual evidence and gap map has been developed by several agencies (see Saran and White, 2018) [26], most notably by the International Initiative for Impact Evaluation (3ie) [27] in the field of international development and subsequently adopted and adapted to a wider a range of sectors through the Campbell Collaboration. These include, for example, transport [28], youth violence, disability (Saran et al. [29]), employment (Campbell et al. [30]), and health and elder abuse [31] (Table 1).

### Suggested approaches for distinguishing between mapping reviews and mapping reviews with EGMs and scoping reviews

The emergence of two terms (scoping and mapping) to describe approaches that have much in common in terms of their objectives and methods suggests that the terms used will be shaped more by the academic background of the researcher than by inherent differences in the approaches.

Currently, as we have shown, there are many instances where “mapping and scoping” are used interchangeably. We argue, in this paper, that while there is considerable overlap between these approaches, there is value in creating a distinction between scoping reviews, mapping reviews, and evidence gap maps. They also could be considered complementary, and a review may have elements of both “mapping” and “scoping.” Each approach, within this family of “broad approach and exploratory reviews” however has a shared objective which is to overview a wider research/topic area, rather than to address a tightly focused question. The methods thereafter diverge in part to address the nature of the research question, the research objectives, the topic area, the depth required for the data extraction, and the expertise of the review team.

**Fig. 1:**
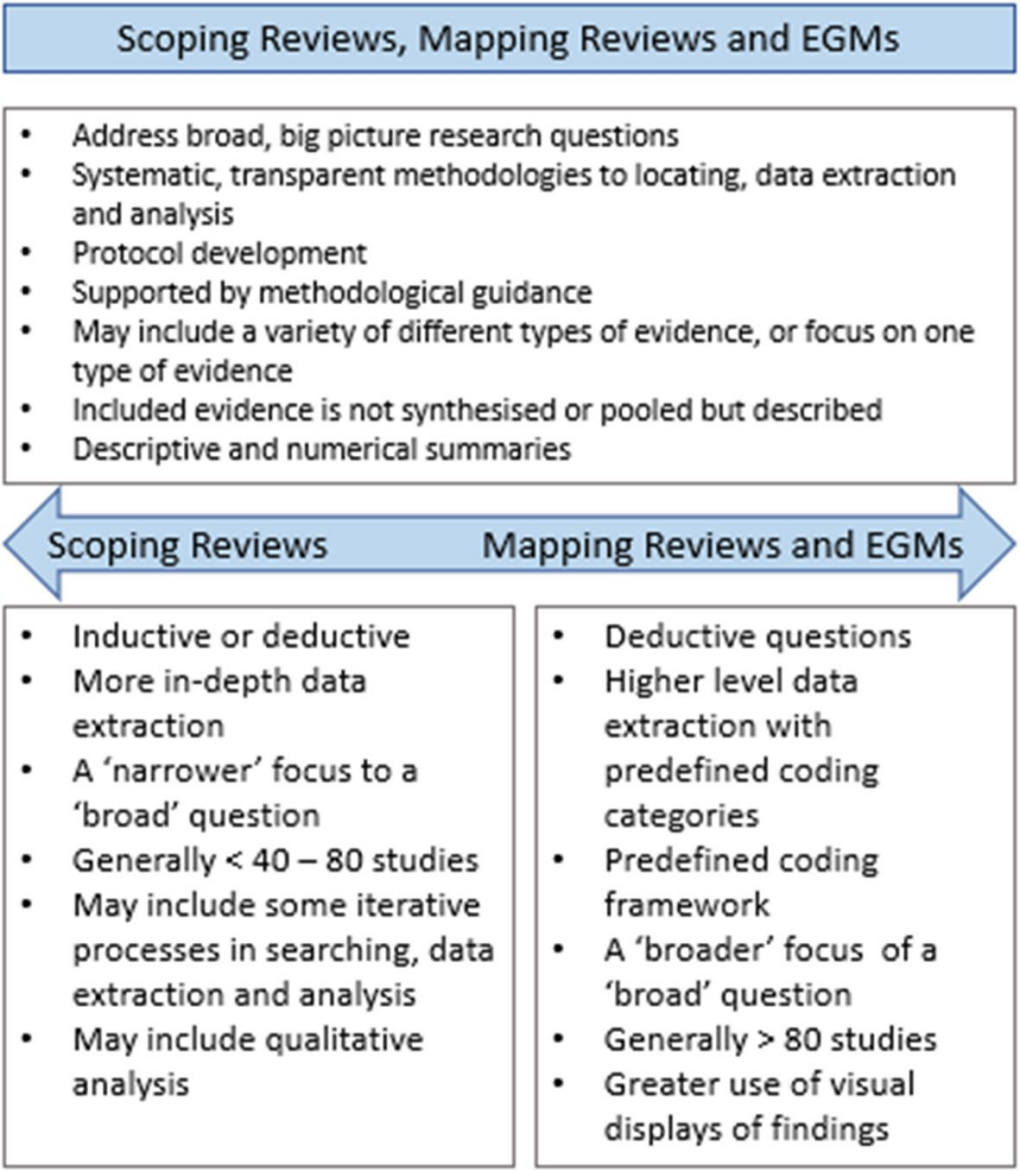
The Big Picture review family (commonalities and differences in approaches)

We propose that a useful distinction is to see mapping, scoping, and EGMs sitting within the same family of types addressing broad questions but sitting on a spectrum in some of their underpinning epistemologies, concepts, and hence objectives (Fig. 1).

This is illustrated in the figure below:

#### Scoping review

These review types have been variously defined and described in the literature as described above. To address the confusion in this field, a recent formal definition of scoping reviews has been proposed, describing scoping reviews as follows:

It is a type of evidence synthesis that aims to systematically identify and map the breadth of evidence available on a particular topic, field, concept, or issue, often irrespective of source (i.e., primary research, reviews, non-empirical evidence) within or across particular contexts. Scoping reviews can clarify key concepts/definitions in the literature and identify key characteristics or factors related to a concept, including those related to methodological research [32].

They can be more exploratory than mapping reviews and EGMs, not requiring an a priori set of codes in order to describe data and may draw upon a range of sources of information *(i.e.,* *primary research, reviews, non-empirical evidence) within or across particular contexts.*The approach can be more iterative, inductive, or deductive [32]. The nature of the “cataloging” and coding may be in response to what is found within the literature or using pre-defined categorization codes. Scoping reviews can also be used to identify concepts and clarify terms in the literature. In contrast to a mapping review where the process of coding is predefined. Within a scoping review, the data extracted may be textual and descriptive, allowing for example an analysis of concepts and categories using simple content analysis. It may include both predefined coding and also exploration of themes (for example, Kelly-Blake et al. 2018 [33]). In contrast, along a continuum, mapping reviews will address broader questions, use predefined coding, and adopt less in-depth data extraction.

**Table 1:** Summary of the different roots and institutions that use mapping and scoping reviews

	**Scoping review**	**Mapping review**	**EGM**
**Academic roots**	Social sciences Arksey & O’Malley 2005 [23] Levac 2010 [24] Khalil et al. 2016 [4] Peters et al. 2020 [12]	Public health, Biomedical sciences, Environmental science James 2016 [18]	International Development 3ie Snilstveit et al. 2013 [27] Saran & White 2018 [26]
**Research concepts**	*Inductive and *Deductive	Deductive	Deductive, inductive
	*Configurative *Aggregative	Aggregative	Aggregative
**Guidance for methods (and reporting)**	JBI (PRISMA ScR) [5–8]	SCIE, Campbell Collaboration (PRISMA ScR)	Guidance: Campbell White et al. [34]
**Identifies gaps in the research**	Yes	Yes	Yes—using a pre-specified framework
**Visual and interactive web-based gap map**	No—but may contain tables and diagrams within text	No—but may contain within text tables and diagrams—and may be produced with an EGM	Yes

^*^Aggregative synthesis: where the synthesis is predominantly aggregating (adding up) data to answer the review question

^*^Configurative synthesis: where the synthesis is predominantly configuring (organizing) data from the included studies to answer the review question

Aggregation and configuration fall on a continuum and all reviews are likely to both aggregate and configure data to some extent [35]

^*^Deductive reasoning: a pre-existing theory or framework that must be tested

^*^Inductive reasoning: an unknown theory or framework that needs to be developed

#### Mapping review

Mapping reviews are also a transparent, rigorous, and systematic approach to identifying, describing, and cataloging evidence and evidence gaps in a broader topic area. They are to collate, describe, and catalog the available evidence relating to the question of interest [18]. They aim to answer the question “what do we know about a topic,” or “what and where research exists on a particular area.” A mapping review typically extracts only descriptive information about the studies and applies predefined codes (high level data). In this sense, they may be informed by an “aggregative” logic. A mapping review may or may not be accompanied by an EGM but provides visual summaries in the form of tables and graphs within the text [36]. These types of reviews may well have broader focus than a scoping review, with more limited data extracted from the included papers.

#### Evidence and gap maps

Evidence and gap maps are described as “a systematic presentation of all relevant evidence of a specified kind for a particular sector, sub-sector, or geography”. Evidence and gap maps (EGMs) are a systematic evidence synthesis product which displays the available evidence relevant to a specific research question. EGMs consist of primary dimensions or framework (rows and columns) and secondary dimensions or filters, enabling exploration of the map using a particular focus (e.g., looking at particular populations or study designs). It creates a visual, web-based, and interactive output [34].

This type of evidence synthesis generally uses a deductive approach with a pre-specified framework to classify the data and identify gaps in the literature. However, if no suitable framework is available, then the research team can develop their own by drawing on the range of resources, such as strategy documents, policy document, and funder reports. This is one of the major differences between mapping with an EGM review and scoping reviews (for the latter, an inductive or deductive approach may be used to identify relevant data elements so the framework for classification of the data and identification of gaps does not need to be pre-specified). Evidence gap maps may accompany a mapping review as a visual representation of the included studies or can stand independently from an accompanying mapping review.

### Purpose

All three of these approaches are characterized by seeking to address a broader topic area rather than a specific intervention or exposure. They are an appropriate tool if the research question is one in which multiple dimensions need to be considered, for example, multiple interventions, outcomes, or types of evidence. They do not aim to synthesize data but rather describe, categorize, and catalog findings. They aim to do so by applying defined methods to ensure transparency and rigor in the process of identifying, screening, data extraction, and interpreting findings. By addressing a broad topic area these approaches support the following purposes [3]:

Knowledge generation to support broad research questions and objectives such as the following:

- What types of evidence are available in a given field?
- How are concepts or definitions used within the literature?
- How and where research is conducted on a certain topic?

The type of broad research question will inform the choice of approach. Scoping reviews are more likely to address open questions and the concepts may be emergent such “how is a key term used within the literature,” in contrast a mapping review may address more closed questions such as “how often the key term is used within the literature and within which population groups.” An evidence gap map will similarly address a closed question, for example, “is the term used in the following types of population group: children, adolescents, older people, and people with chronic conditions.”

Scoping reviews can provide an approach that allows exploration and clarification of key concepts and definitions within the literature, as well as how research is undertaken. As this approach does not require predefined categories, it allows for more descriptive data extraction. Often the question will be narrower than in a mapping review, allowing a greater depth of exploration of the included studies.

These approaches enable a better understanding is gained of phenomena by seeing it within a wider context. Olson et al. 2021 [37] uses the allegory of the blind monks who examine the elephant, where close inspection of one part of the whole means that meaning is lost. A complete picture is needed to really understand what the elephant is. It is clear, when seeking to operationalize what is meant by a “broad” topic area that perspective matters. For a cell biologist, the cell nucleus might be a broad topic, which a single country might be too narrow a perspective for the geographer. Understanding this unique feature of “Big Picture” reviews is perhaps easier when seen in contrast to the approach used in a systematic review examining the effectiveness of a single intervention. A Big Picture review question will look at multiple interventions or exposures and multiple outcomes or effects, seeking not to synthesize but to describe (Table 2).

### To provide a foundation for guiding future research priorities and decisions by identifying available evidence and gaps in research

Mapping reviews and EGMs incorporate a framework that is generated during development of the protocol—it is this framework which guides the development of the data extraction tool or coding tool. This framework becomes the “map” against which existing evidence is plotted.

Identifying research gaps is often a stated part of all types of research; indeed, implications for “research and practice” are an expected part of all health and social care-related research. Identifying research gaps is often a primary purpose of scoping, mapping, and mapping reviews with EGMs more than other types of review design. In particular, mapping reviews with or without evidence gap maps address this purpose with a transparency and rigor that is unique.

Evidence and gap maps aim to enable evidence to be located, both by showing what is there but also in demonstrating knowledge gaps. In order to identify knowledge gaps, an EGM begins by developing the framework against which the evidence is plotted. The development of the framework adheres to the following principles. Firstly, it may be constructed using an existing, widely accepted international typology for either interventions, exposures, or outcomes. Secondly, if no suitable framework is available then the research team may draw on a range of resources including consultation with stakeholders and relevant published theories to ensure the comprehensiveness of the framework. Without such a structure, the gaps are not identified in a systematic way, but rather inferred and chosen by the review authors (no doubt well informed) but nevertheless influenced by their own perspectives and bias. This may be particularly apparent where a review is undertaken to pave the way for further primary research by the same team. Review teams could be strongly invested in identifying their own planned research as the “research gap.”

Evidence gap maps are a systematic approach to identifying the evidence and in particular—its gaps. No other review methodology has developed a systematic approach to identifying gaps in the evidence with this level of rigor and transparency. A limitation of the approach is that it only charts what is known and does not allow a more exploratory approach that may be employed in a scoping review.

Mapping and mapping reviews with EGMs aim to describe the state of evidence for a question or topic. The review questions may therefore be open framed and broad. However, the question can be close framed and narrow. Key elements of the question can be formulated by a framework such as PO (population, outcome). For an EGM, the objectives are formalized in the framework which defines the scope of the map [34].

### To inform policy decisions, where an overview of an area may be more helpful than specific questions about specific types of interventions

Mapping (with or without an EGM) and scoping reviews often have pertinence for policy makers as they are able to cover the breadth of science often needed for policy-based questions; however, it needs to be remembered that the mapping approaches do not synthesize the findings and not include quality or risk of bias appraisal. These factors may limit their value to support some types of policy decisions. However, a mapping review with an accompanying EGM can take users to the research papers and facilitate the ready location of relevant evidence. An EGM can take users to the research papers and facilitate the ready location of relevant evidence. One example has been the use of a country evaluation map used by the Office of the Prime Minister of Uganda to identify studies to inform policy work [38]. Similarly, scoping reviews can inform policy and further research through identifying the available literature pertaining to a particular topic, along with clarifying key concepts and definitions.

### As a stepping stone to building the evidence architecture

Evidence mapping and EGMs may be used as a first step towards the generation of evidence-based decision-making products, such as guidance, checklists, and online decision-making tools [39]. Maps will identify the (i) existing reviews which are suitable to use a basis for guidance, etc., (ii) where there are clusters of primary studies but no review so reviews may be commissioned in priority areas to inform guidance, etc., and (iii) important policy areas in which evidence is missing. To serve this purpose, the map should be regularly updated (maintained).

## Discussion

While the literature is inconsistent in its definitions of these types of reviews, and different reviews use different terminology to describe methods that appear very similar, many of these differences reflect the different research traditions and adoption of terms within organizations undertaking these types of syntheses. We argue that there is value in having these distinct terms to describe the different approaches within this family of broad review types. Scoping reviews allow a more inductive, in-depth approach with, including fewer included studies and a greater level of data extraction compared with mapping reviews. Mapping reviews and evidence gap maps address more closed questions, with pre-specified items defined and code-able when contrasted with scoping reviews. Evidence gap maps offer a visual, interactive output for users to locate evidence. The predefined framework offers a rigor to locating gaps in the existing literature and displaying these differences which is unique to these approaches.

This proposed new “Big Picture” review family within evidence synthesis contributes to the wide array of possible approaches to synthesizing literature. This multitude of choice presents challenges in selecting the correct evidence synthesis methodology. One tool that has been developed to assist in the appropriate selection of a method is the “right review” tool (https://whatreviewisrightforyou.knowledgetranslation.net/). The tool enables researchers to answer a series of simple questions regarding the type of research questions they are undertaking for their review and selects an appropriate type of review based on their answers to the questions. The tool currently includes 41 different types of evidence synthesis methods [40].

A recent development has been changes made to the SR Toobox (http://systematicreviewtools.com/index.php) to include searching for tools to support different review types, as well as for different stages of the review. The Big Picture review family is increasingly well supported by methodological guidance and automation tools to support the process of undertaking high quality systematic reviews.

**Table 2:** Examples of review aims

**Type of approach**	**Aim**
Scoping review	To report in detail the methodology employed to identify relevant theories and provide a list of agreed criteria for judging the quality of theories (Davis et al. 2015) [41] To document and describe the evidence base relating to stakeholder involvement in systematic reviews and to use this evidence to describe how stakeholders have been involved in systematic reviews (Pollock 2018) [42]
Mapping review	A mapping review of research on gambling harm in three regulatory environments (Baxter et al. 2019) [43]
EGMs	To identify what has been published on micronutrients and depression and identify gaps in the evidence and collections suitable for meta-analysis. (Campisi et al. 2020) To identify intergenerational interventions and the social and mental wellbeing outcomes that have been measured in their evaluation. (Thompson-Coon et al. 2022)

The existing guidance for the conduct and reporting of scoping reviews also applies to mapping reviews (JBI). Further development is needed in the methods of preparing a coding framework, particularly when the mapping review will also include the development of an interactive EGM. Current models of good practice exist; however, current guidance and reporting standards are limited.

## Conclusion

This commentary details and describes some of the broad approaches within the evidence synthesis toolkit, specifically scoping reviews, mapping reviews, and EGMs. We have identified similarities and differences, based on our expert experience, between these reviews. We propose grouping them as a family of evidence synthesis to address broad research question and objectives. In so doing, we advocate that adherence to the principles of rigor and transparency that give users of evidence synthesis confidence in the reliability of the results of the review.

## Appendix

Useful resources: https://guides.temple.edu/c.php?g=78618&p=4156607 and https://wiki.joannabriggs.org/display/MANUAL/11.2+Development+of+a+scoping+review+protocol.
