# Correction: Mapping reviews, scoping reviews, and evidence and gap maps (EGMs): the same but different— the “Big Picture” review family

**DOI:** 10.1186/s13643-023-02224-2

**Published:** 2023-04-01

**Authors:** Fiona Campbell, Andrea C. Tricco, Zachary Munn, Danielle Pollock, Ashrita Saran, Anthea Sutton, Howard White, Hanan Khalil

**Affiliations:** 1 Population Health Sciences Institute, Newcastle University, Newcastle, UK; 2 Knowledge Translation Program of the Li Ka Shing Knowledge Institute, St. Michael’s Hospital, University of Toronto in the Dalla Lana School of Public Health & Institute of Health Policy, Management, and Evaluation, Toronto, Canada; 3 JBI, Faculty of Health and Medical Sciences, University of Adelaide, Adelaide, Australia; 4 International Development Coordinating Group, Campbell Collaboration, Oslo, Norway; 5 ScHARR, University of Sheffield, Sheffield, UK; 6 Evaluation and Evidence Synthesis, Global Development Network, New Delhi, India; 7 School of Psychology and Public Health, Department of Public Health, La Trobe University, Melbourne, Australia

**Correction: J Ovarian Res 12, 45 (2023)**

10.1186/s13643-023-02178-5

Following publication of the original article [1], the authors identified an error in the author name of Zachary Munn and Danielle Pollock and affiliation of Fiona Campbell.

The incorrect author names are: Zacchary Munn, Dannielle Pollock

The correct author name is: Zachary Munn, Danielle Pollock

Affiliation 2 has been removed from author Fiona Campbell.

The author group has been updated above and the original article [1] has been corrected.
